# Determination of production capacity for open-pit coal mines under uncertainty: A model based on economies of scale

**DOI:** 10.1371/journal.pone.0312130

**Published:** 2025-01-09

**Authors:** Shuai Wang, Bo Cao, Runcai Bai, Guangwei Liu

**Affiliations:** College of Mining, Liaoning Technical University, Fuxin, Liaoning Province, People’s Republic of China; Akita University: Akita Daigaku, JAPAN

## Abstract

The determination of optimal production capacity for open-pit mines is influenced by various economic and technical factors and is highly susceptible to uncertainties. To effectively address the impact of uncertainty on capacity planning, this study develops a production capacity planning model for open-pit coal mines, based on the theory of economies of scale and incorporating multiple uncertainty constraints. A novel method is proposed to quantify and propagate the uncertainties of key capacity control factors, alongside a comprehensive uncertainty analysis framework for total revenue. Using a large-scale open-pit mine as a case study, the relationships and calculation tables for the uncertainties of key control factors are provided. Based on this, a mathematical expression for total revenue under the influence of multiple uncertainties is formulated, and the optimal capacity range is calculated. The application of the model is demonstrated through this case study, revealing that the uncertainties in production costs and coal prices are (-13.05%, +27.68%) and (-10.79%, +10.79%) respectively. Subsequently, risk-tolerant capacity planning recommendations are proposed, effectively mitigating the impact of uncertainties on production capacity determination.

## 1. Introduction

In open-pit coal mine design, planning an appropriate production capacity is of utmost importance, as it directly affects the mine’s return on investment [1, 2]. Production capacity is related to the mine’s technical conditions and is influenced by geological conditions of the orebody, reserve size, and market environment [3]. Capacity planning is a strategic process aimed at determining the capacity needed to meet forecasted demand, production scheduling, supply chain planning, and inventory management [4]. The determination of optimal production capacity can be divided into two key steps [5]: (1) Establishing the technical upper and lower limits based on mining methods and equipment, and (2) Determining a reasonable production scale through economic analysis. Traditional methods for optimizing production scale can be classified into three categories: (1) Empirical formula methods, such as the Taylor Formula [6], (2) Economic analysis methods, which include the method of reasonable service life based on national economic considerations and the optimization of economic indicators [7, 8], and (3) Comprehensive analysis methods, such as fuzzy comprehensive evaluation and grey multi-objective decision-making. Among these, economic analysis methods are the most commonly used in production scale optimization due to their broad applicability and high reliability.

The ultimate goal of open-pit mining is to derive the economic value of mineral resources [9, 10], with total revenue directly linked to mineral prices and production costs [11]. Due to the extensive extraction areas of open-pit mines, block models are typically used to estimate mineral resource reserves [12, 13], which are then used to calculate stripping costs and other economic parameters [14]. Determining an optimal production capacity requires a comprehensive analysis that integrates mining technology, equipment levels, economic returns, and management practices [15, 16]. Given that uncertainty is an inherent characteristic of these factors [17, 18], decision-making often results in ambiguity and imprecision. Therefore, it is essential to appropriately quantify uncertainty in the production design process [19].

The theoretical framework for studying uncertainty was established by Liu [20], designed to measure and model the degree of human belief uncertainty. Fundamentally, uncertainty is an attribute of risk analysis and can sometimes be reduced through further measurement or investigation [21]. In the process of uncertain risk assessment, decision-makers exhibit differentiated risk preferences, and effectively expressing and handling this heterogeneity in individual risk preferences has been a central focus in the field of uncertain risk analysis [22, 23]. Traditional methods for uncertainty analysis are often computationally expensive, especially methods such as Monte Carlo simulations (MCM) or Latin Hypercube Sampling (LHS) [24, 25]. In recent years, numerous improved methods have been developed to reduce computational costs and enhance efficiency [26], such as prospect theory [27] fuzzy theory [28], and hybrid methods [29]. Among these, bootstrap simulation, introduced by Efron [30], was designed to estimate the confidence intervals of statistics using numerical methods [31]. One key advantage of bootstrap simulation is its ability to estimate confidence intervals even in cases where mathematical solutions may not exist [32]. Therefore, in this study, the bootstrap simulation method is employed to quantify the uncertainties in mineral prices and production costs, and subsequently, the determination of optimal production capacity is investigated under the influence of these uncertainties.

Simultaneously, when studying the impact of uncertainties on production capacity planning, both the maximization of economic benefits and minimization of risks must be considered. Scholars have conducted extensive research on the influence of uncertainties on production capacity. Levinson and Dimitrakopoulos developed a synchronous stochastic optimization framework for long-term production scheduling in mine complexes, aiming to enhance product value while minimizing technical risks associated with uncertain material supply [33]. Brika et al. proposed an optimization method for long-term production planning in open-pit mines, accounting for uncertainty in mineral supply and investment decision-making for multiple processing streams [34]. Boachie highlighted the importance of dynamic economic modeling and ongoing geotechnical assessments [35] Liu et al. analyzed the relationship between working face length, annual advance rate, and production capacity, constructing a functional relationship model for production capacity [1]. Khorolskyi et al. defined optimal production from the perspective of production activities and the correlation of operational resources [36]. Although these studies have made significant contributions to addressing the impact of uncertainties on mine production capacity, they mainly focus on production scheduling, technical risks, and resource correlations, without systematically analyzing how uncertainties specifically affect production capacity. As open-pit mines function as large-scale commodity production enterprises [15], their production processes typically follow the theory of economies of scale [37]. By analyzing the cost-benefit relationships under different production scales, enterprises can flexibly adjust their production scale to respond to uncertainties. Therefore, applying economic analysis based on economies of scale theory may be more effective in managing uncertainty.

Based on this, the present study starts from the concept of marginal revenue in the economies of scale curve and constructs a production capacity planning model for open-pit mines under uncertainty by analyzing the relationship between total revenue and variable factors. By identifying and describing key uncertainty factors, the uncertainties affecting production capacity planning are quantified. Through risk management techniques, the uncertainty in total revenue is further analyzed, and a method for determining optimal production capacity that incorporates risk is proposed. Using actual production data from a specific open-pit mine, the upper and lower limits of production capacity are determined. Principal component analysis (PCA) is then employed to identify key factors affecting costs. After analyzing the uncertainties in coal prices and total costs, a risk-controlled optimal production capacity range is proposed to guide capacity planning for open-pit mines.

## 2. Methodology

### 2.1 Economies of scale theory

**Fig 1:**
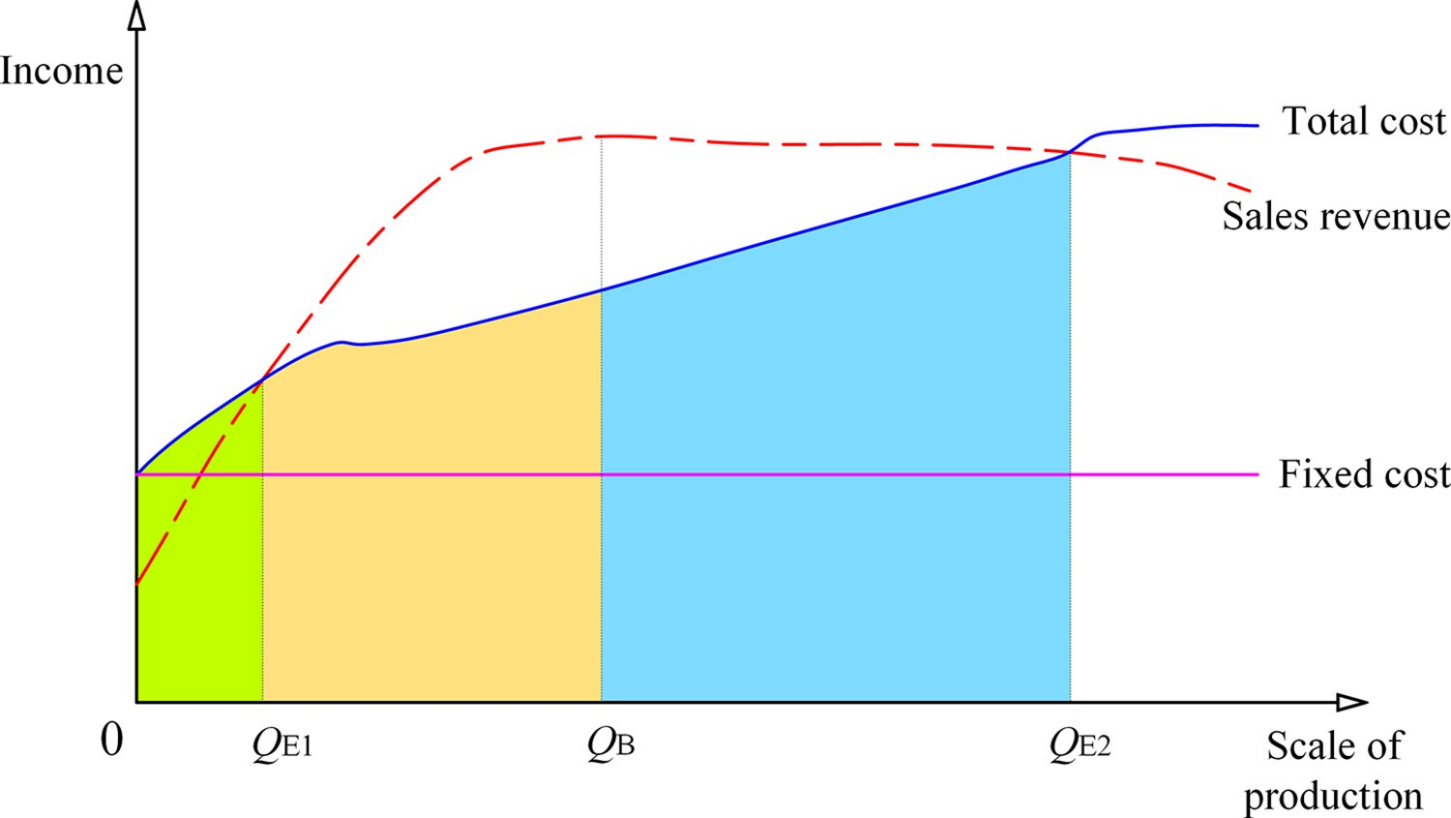
Scale effect curve.

Economies of scale theory primarily examines the relationship between the scale of economic activities and their long-term costs. When the average cost of a product decreases as production scale increases, it is referred to as economies of scale. Conversely, if the average cost rises with an increase in scale, it is termed diseconomies of scale. Research on determining economies of scale focuses on analyzing the impact of production scale on costs and profits, which involves factors such as sales revenue, total cost, and profit. This relationship is illustrated in Fig 1.

As shown in Fig 1, when the production scale reaches *Q*_*E*1_ or *Q*_*E*2_, the enterprise breaks even; when it exceeds *Q*_*E*2_, the enterprise begins to lose money; when the production scale is between *Q*_*E*1_ and *Q*_*B*_, the scale benefit of the enterprise increases, meaning income increases more than production scale; when the production scale exceeds *Q*_*B*_, the income of enterprise-scale shows a decreasing trend. The range from *Q*_*E*1_ to *Q*_*B*_ is the economies of scale.

In the theory of economies of scale, in addition to using financial indicators such as net present value to measure the rationality of production scale, the concepts of “marginal cost” and “marginal income” are usually used. The marginal cost is the value by which the total cost changes with each increase or decrease of one product unit, and the marginal income is the value by which production increases the total income for each additional unit of product sold, that is:

$$C_{M}=C^{\prime }(Q)=\underset{\Delta Q\to 0}{\lim}\frac{\Delta C}{\Delta Q}=\underset{\Delta Q\to 0}{\lim}\frac{C(Q+\Delta Q)-C(Q)}{\Delta Q}$$ (1)

$$R_{M}=R^{\prime }(Q)=\underset{\Delta Q\to 0}{\lim}\frac{\Delta R}{\Delta Q}=\underset{\Delta Q\to 0}{\lim}\frac{R(Q+\Delta Q)-R(Q)}{\Delta Q}$$ (2)

where *C*_*M*_ is the marginal cost, yuan/t; Δ*C* is the increment of total cost, yuan; Δ*Q* is the increment of output, t;*R*_*M*_ is the marginal income, yuan/t; and Δ*R* is the total income increment, yuan.

According to the law of diminishing marginal income, diminishing marginal income occurs under certain conditions. In view of limited resources, the scale of the mine has a certain limit, beyond which the total cost increases considerably while income decreases accordingly. From a reasonable economic perspective, the scale of the mine should be limited to avoid diminishing returns. Therefore, the reasonable scale of economy should conform to the law of marginal income, that is, it should avoid the period of diminishing marginal income.

In summary, the theory of economies of scale can be used to relatively easily determine the upper and lower limits of optimal production capacity. However, when influenced by uncertainties such as coal price fluctuations, resource conditions, and mining technology levels, the impact of economies of scale on production capacity limits becomes more complex.

(1) Coal Price Fluctuations:

When coal prices decline, the cost advantages brought by economies of scale are weakened, leading to a lower upper limit and a higher lower limit. Conversely, when coal prices rise, the increase in expected revenue raises the upper limit and lowers the lower limit. In situations of market price instability, larger production scales also imply greater risks and higher inventory pressures, thereby reducing the upper limit.

(2) Changes in Resource Conditions:

When resource conditions improve, expected revenues increase, leading to a higher upper limit and a lower lower limit. However, when resources near depletion, the actual extractable volume decreases, and a larger production scale represents a higher risk, causing the upper limit to decline.

(3) Technological Advancements:

The application of new technologies will reduce unit production costs, raising the upper limit. Conversely, low equipment efficiency may result in a lower lower limit.

### 2.2 Production capacity planning model

The construction of a production capacity planning model should fully account for the current mining conditions to determine the technically feasible upper and lower limits of production capacity. Based on the theory of economies of scale, the model establishes functional relationships between production volume and cost, as well as between production volume and mineral prices, ultimately solving for the economically feasible optimal production capacity range.

#### 2.2.1 Determination of upper and lower limits of production capacity

The planning and decision-making of production capacity in open-pit mining must emphasize the influence of variations in orebody characteristics and the specific features of open-pit mining processes. The objective is to ensure both technical feasibility and economic rationality. Therefore, the upper and lower limits of production capacity are influenced by factors such as resource reserves, technical conditions, and economies of scale.

*2*.*2*.*1*.*1 Upper limit scale determined by resource reserves*. A reasonable service life should maximize the efficiency of equipment and engineering facilities while minimizing investment and production costs. According to the widely recognized empirical equation of the relationship between the economic life of open-pit mines and ore field reserves [5], the design capacity and life are calculated by Eq (3):

$$T=\frac{Q}{A}=6.5\sqrt[{4}]{Q}(1\pm 0.2)$$ (3)

*2*.*2*.*1*.*2 Upper limit scale considering technical constraints*. (1) Maximum production scale according to mining intensity

The production scale of open-pit mines determined by the intensity of mining technology is as follows [2]:

$$A=Lv\gamma h\frac{1-t}{1-f}$$ (4)

where *L* is the length of the working line in the open-pit mining area, m; *v* is the progress of the mine, m/a; *h* is the average thickness of coal seam mined in the mine, m; *γ* is the bulk density of raw coal mined in an open-pit mine, t/m^3^; *t* is the loss coefficient of coal seam mining; *f* is the gangue mixing coefficient.

(2) Maximum production scale according to main production links

Open-pit mine production is a series system composed of the loose crushing of ore and rock, mining and loading, transportation, dumping, and other links [14]. When verifying the production scale of the open-pit mine, the minimum value of the link capacity is taken, that is,

$$A=min\{P_{d}+V_{u},P_{l},P_{h},P_{s}\}$$ (5)

where *P*_*d*_ is the drilling and blasting link capacity, m^3^/a; *V*_*u*_ is the annual planned excavation volume of loose materials without blasting, m^3^/a; *P*_*l*_ is the mining and loading link capacity, m^3^/a; *P*_*h*_ is the transport link capacity, m^3^/a; *P*_*s*_ is the dumping link capacity, m^3^/a.

*2*.*2*.*1*.*3 Lower limit scale to ensure mine benefit*. As the mine should be profitable or at the minimum run at zero loss, the minimum scale to ensure the minimum benefit of the mine is [37]:

$$A_{min}\geq \frac{K_{1}\cdot C_{d}}{P-K_{2}C_{o}}$$ (6)

Where, *A*_*min*_ is the lower limit scale to ensure the minimum economic benefit, 10^4^ a, *C*_*d*_ is the fixed total cost, yuan/a; *K*_1_ is the coefficient that considers the effect of scale on fixed costs, taking 0.9; *C*_0_ is the variable unit cost, yuan/t; *K*_2_ represents the adjustment coefficient, which partly proportional and taken as 0.95; *P* is the price of coal, yuan/t.

#### 2.2.2 Construction of relationship between production capacity and mineral price function

As coal prices are mainly affected by the coal market, coal price fluctuations will affect the sales of coal, creating a feedback on coal prices. In neoclassical economics, the cobweb model introduces the factor of time change; investigates the interaction among demand, supply, and price in different periods; and analyzes the law of product price fluctuation with a long production cycle. The theory postulates that producers always determine the current output according to the previous price, and under the equilibrium of supply and demand, the current output will affect the current price.

The basic assumption of the cobweb model is that the current output *Q*_*t*_ of the commodity is determined by the previous price *p*_*t*-1_, that is, the supply function is *Q*_*t*_ = *f*(*p*_*t*-1_); the current output *Q*_*t*_ of the commodity determines the current price *p*_*t*_, that is, the demand function is *p*_*t*_ = *f*(*Q*_*t*_). This paper focuses on the relationship between production capacity and price. To ensure the effectiveness of the model, the following assumptions are made:

(1) relevant stakeholders in the coal market only consider benefits and costs, and policy factors are considered only in extreme cases;

(2) the total consumption of coal in the coal market is fixed, and the total demand is less than the maximum supply;

(3) when analyzing the coal market separately, competition exists among different coal enterprises, but overall, no obvious difference exists between coal enterprises.

**Fig 2:**
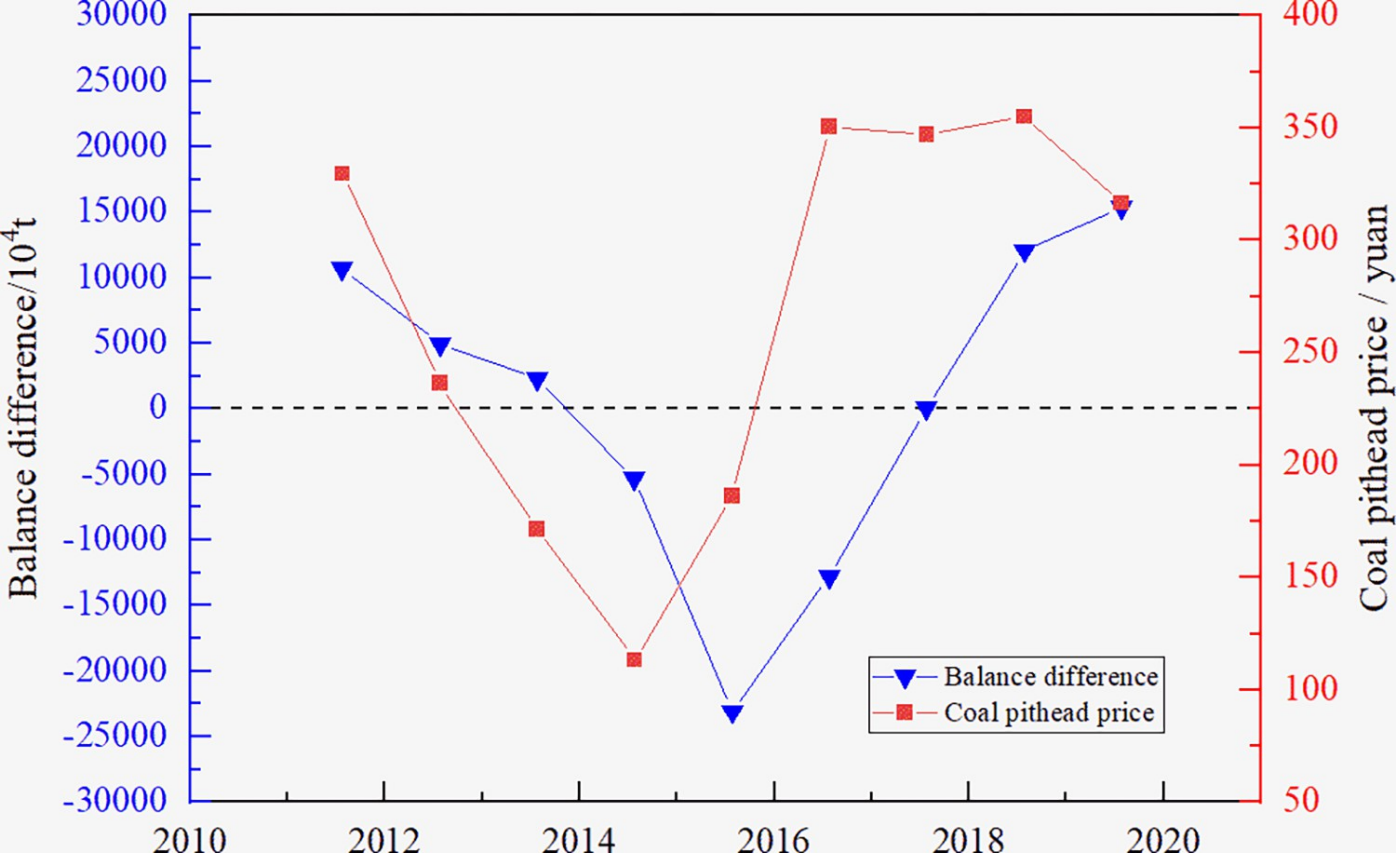
Relationship between supply-demand and coal price.

According to the cobweb model dynamic theory, the last period price determines the current period output, the current period output determines the current period price. This theory is applied to construct the coal supply and demand analysis model using relevant data on coal production, coal import and export, total coal consumption, and coal pit price from 2012 to 2020. Considering the characteristics of open-pit coal production, the relationship between coal supply and demand and price is described using the balance difference and price of a raw coal pit, as shown in Fig 2.

As shown in Fig 2, when the balance difference is greater than 0, the supply exceeds the demand, resulting in a drop in the coal price in the next period, and the coal production enterprises tend to reduce the production scale. When the balance difference is less than 0, the supply is less than the demand, which leads to the rise in coal prices in the next period, and the enterprises tend to expand the production scale.

Considering the hypothesis of the cobweb model, we assume that the relationship between the current production scale and the previous coal price is *Q*_*t*_ = *a*_1_+*b*_1_*P*_*t*−1_+*e*_1_, and the relationship between the current price and current output is *P*_*t*_ = *a*_2_+*b*_2_*Q*_*t*_+*e*_2_. The cobweb model of coal output is obtained using the data in Fig 2 and the method of least squares. The results of the model are presented in Table 1.

**Table 1:** Estimation results of cobweb model in coal market.

Model equation	*R* ^2^	*Adj R* ^2^	*F* value	*P* value
*Q*_*t*_ = 355223.7+15765.6*P*_*t*−1_	0.6214	0.5583	9.8483	0.0201
*P*_*t*_ = 38.6637+0.000757*Q*_*t*_	0.03151	0.1068	0.2278	0.6477

#### 2.2.3 Construction of production capacity—cost function relationship

To explore the behavior of the variation between the total cost and annual output of the mine, the general expression of the functional relationship is constructed as shown in Eq (7).


$$C_{z}(Q)=a+bQ+cQ^{2}$$ (7)

According to the possible relationship between total cost, fixed cost, and variable cost, Eq (7) is expressed in three possible forms, as shown in Eq (8).


$$\{\begin{array}{l} b=0,c\neq 0,C_{z}(Q)=a+cQ^{2}\\ b\neq 0,c\neq 0,C_{z}(Q)=a+bQ+cQ^{2}\\ b\neq 0,c=0,C_{z}(Q)=a+bQ \end{array}$$ (8)

In Eq (8), the value of *a* depends on the mining conditions of different mines and is selected according to experience; the value of *c* is a variable cost parameter, which is related to the quality of resources, in which the *c* of high-quality ore resources is relatively small, whereas that of ordinary ore resources is relatively large. The functional relationship between the mine production cost and the annual output of the mine can be established by determining the values of *a*, *b*, and *c* in Eq (8).

### 2.3 Analysis of the relationship between total cost and key variable costs

#### 2.3.1 Principal component analysis (PCA)

A key issue in constructing a production capacity planning model is identifying the principal factors that affect production capacity. Principal Component Analysis (PCA) is a commonly used method for determining these principal factors. This involves systematically collecting data on mining production costs and establishing the original sample matrix *X* for mining production costs.


$$X=\left(X_{1},X_{2},\cdots ,X_{P}\right)={\left(x_{kj}\right)}_{n\times p}=\{\begin{array}{cccc} x_{11} & x_{12} & \cdots & x_{1p}\\ x_{21} & x_{22} & \cdots & x_{2p}\\ \vdots & \vdots & \cdots & \vdots \\ x_{n1} & x_{n2} & \cdots & x_{np} \end{array}\}\left(k=1,2,\cdots ,n;j=1,2,\cdots ,p\right)$$ (9)

PCA is a statistical method that considers the relationship between indicators and uses dimensionality reduction to convert multiple indicators into a few unrelated indicators, making further research simple. The PCA method can be broken into the following steps:

(1) Standardize the original data. Standardization Eq (9) is used to standardize the original sample matrix *X*, and the standardization matrix X* is obtained, as shown in Eq (10).


$$x_{kj}^{*}=\frac{x_{kj}-\bar{x_{j}}}{\sqrt{S_{jj}}}\left(k=1,2,\cdots ,n;j=1,2,\cdots ,p\right)$$ (10)

$$X^{*}={\left(x_{kj}^{*}\right)}_{n\times p}=\{\begin{array}{cccc} x_{11}^{*} & x_{12}^{*} & \cdots & x_{1p}^{*}\\ x_{21}^{*} & x_{22}^{*} & \cdots & x_{2p}^{*}\\ \vdots & \vdots & \cdots & \vdots \\ x_{n1}^{*} & x_{n2}^{*} & \cdots & x_{np}^{*} \end{array}\}\left(k=1,2,\cdots ,n;j=1,2,\cdots ,p\right)$$ (11)

(2) Calculate the correlation coefficient matrix.


$$R={\left(r_{ij}\right)}_{p\times p}=\{\begin{array}{cccc} r_{11} & r_{12} & \cdots & r_{1p}\\ r_{21} & r_{22} & \cdots & r_{2p}\\ \vdots & \vdots & \ddots & \vdots \\ r_{p1} & r_{p2} & \cdots & r_{pp} \end{array}$$ (12)

Where $r_{ij}=\frac{S_{ij}}{\sqrt{S_{ii}S_{jj}}}$, and $S_{ij}=\frac{1}{n-1}\sum_{k=1}^{n}(x_{ki}-\bar{x_{i}})(x_{kj}-\bar{x_{j}}),\left(i\neq j;i,j=1,2,\cdots ,p\right)$. The Equation for calculating $\bar{x_{j}}$ is $\bar{x_{j}}=\frac{1}{n}\sum_{i=1}^{n}x_{ij},\left(j=1,2,\cdots ,p\right)$.

(3) Calculate the eigenvalue of the correlation coefficient matrix *R* and its corresponding eigenvector.

(4) The principal component contribution rate and cumulative contribution rate are calculated. The expression of the principal component *Z*_*i*_ is calculated according to Eq (13).


$$Z_{i}=l_{i}X^{T}=l_{i1}X_{1}+l_{i2}X_{2}+\cdots +l_{ip}X_{p}$$ (13)

The contribution rate *α*_*i*_ of each principal component *Z*_*i*_ is calculated according to Eq (14), and the principal components are sorted according to the eigenvalues in descending order of magnitude. The cumulative contribution rate *β*_*q*_ of the first *q* principal components is calculated using Eq (15). When the cumulative contribution rate exceeds 80%, the first *q* principal components approximately contain all the information of the original factors, in which case the number of principal components is determined to be *q*.


$$\alpha_{i}=\frac{\lambda_{i}}{\sum_{i=1}^{p}\lambda_{i}}$$ (14)

$$\beta_{q}=\sum_{i=1}^{q}\alpha_{i}$$ (15)

(5) Calculate the principal components.

Combined with the expression of principal component analysis and its contribution rate, the comprehensive weight of each influencing factor is calculated using Eq (16). The larger the value is, the greater the influence of the change of influencing factors overall, that is, the main controlling factor.


$$r_{j}=\overset{q}{\underset{i=1}{\sum }}\alpha_{i}l_{ij},l_{ij}\geq 0,j=1,2,\cdots ,p$$ (16)

#### 2.3.2 Analysis of the behavior of changes between total cost and the key variable cost

Based on the data of the main factors influencing mine costs, multiple expressions of functional relations in Eq (17) are fitted and analyzed by regression analysis. Eq (18) and Eq (19) are used to determine the goodness of fit *R*^2^ and difference significance *Sig* value corresponding to the five expressions. The values are used to determine the optimal function relationship between fixed cost and variable cost under the influence of the main control factors.

$$Specific\;form:\{\begin{array}{l} Linear\;form:C_{g}^{'}=d_{1}+e_{1}\times C_{b}^{'}\\ Logarithmic\;form:C_{g}^{'}=d_{2}+e_{2}\times ln(C_{b}^{'})\\ Countdown\;form:C_{g}^{'}=d_{3}+\frac{e_{3}}{C_{b}^{'}}\\ Quadratic\;form:C_{g}^{'}=d_{4}+e_{1}\times C_{b}^{'}+m_{4}\times C_{b}^{'2}\\ Exponential\;form:C_{g}^{'}=d_{5}^{\left(e_{5}\times C_{b}^{'}\right)} \end{array}$$ (17)

$$R^{2}=1-\frac{\sum_{i=1}^{n}(y_{i}-{\hat{y}}_{i})}{\sum_{i=1}^{n}(y_{i}-{\bar{y}}_{i})}$$ (18)

where *y*_*i*_ is the sample value, ${\hat{y}}_{i}$ is the fitting value generated by the model, and ${\bar{y}}_{i}$ is the average value of the sample value.


$$Sig=P\{F>F_{0.05}(1,n-2)\}$$ (19)

Thus, the regression analysis method is used to fit and analyze the mine output and the modified mine production cost data, and the function *C*_*z*_ = *f*(*Q*) between the optimal total cost and mine output is selected using the form shown in Eq (17).

### 2.4 Quantification and description of uncertainty

The uncertainty analysis of production capacity planning aims to determine the magnitude or possible range of uncertainty and identify the key sources of uncertainty through the qualitative or quantitative analysis of various sources of uncertainty in the determination of production capacity. The outcome of the analysis provides guidance for production capacity planning.

#### 2.4.1 Uncertainty description

The quantitative uncertainty analysis of factors affecting production capacity is a process of quantifying the uncertainty of parameters and obtaining the probability distribution of uncertainty through statistical analyses or expert judgment to provide basic input for follow-up research. A probability distribution model is usually used to describe the uncertainty of the model input and output, which involves the use of concise mathematical expressions to describe the uncertainty of variable input and output. Common probability distribution models include normal distribution, lognormal distribution, beta distribution, gamma distribution, Weibull distribution, uniform distribution, symmetrical triangular distribution, chi-square distribution, t-distribution, and F-distribution.

#### 2.4.2 Parameter estimation and test

Uncertainty analysis mainly involves point estimation, and the two basic point estimations methods are moment estimation method and maximum likelihood method. The advantage of the moment estimation method is that only the moment of the population is needed; the distribution form of the population is not required. The maximum likelihood estimation method requires the distribution form of the population, and, in general, the solution of the likelihood variance is more complex. Thus, it is necessary to calculate the approximate solution through an iterative operations performed using a computer. For large sample data, the maximum likelihood estimation method has higher reliability and efficiency than those of the moment estimation method. However, for small sample data, the maximum likelihood estimation method does not always produce minimum variance or produces unbiased estimates.

Examples of the commonly used distribution goodness-of-fit test methods in uncertainty analysis include Pearson χ^2^ test, K-S test, and A-D test. From a practical perspective, the K-S test and A-D test are widely used; thus, the above parameters are selected as the test method in this paper.

#### 2.4.3 Quantification of parameter uncertainty

The uncertainty of the relevant parameters affecting the production capacity can be quantitatively analyzed by a probabilistic analysis. The commonly used statistical analysis methods include the classical confidence interval quantization method based on “central limit theory,” a self-developed simulation, or a direct fitting method. Considering that the probability distribution model of the classical confidence interval quantization method must obey the normal distribution and other strict requirements, a more flexible self-expansion is used to simulate the uncertainty of quantitative variables in this paper.

Bootstrap simulation, also known as bootstrapping, is a method proposed by Professor Efron at Stanford University in 1977. It involves resampling from the original data to generate new samples and statistics. The basic idea is to repeatedly resample the data when the entire sample is unknown, creating bootstrap samples, and then analyze these samples to obtain confidence intervals for an estimate. The primary advantage of bootstrap simulation in quantifying input uncertainty is its independence from the type of probability distribution model used to describe input variables, making it more flexible and practical.

Bootstrap simulation exhibits strong robustness in capturing real-world uncertainties, as demonstrated in several aspects:

(1) Multi-Scenario Simulation Capability

Bootstrap simulation can generate a large number of scenarios, systematically covering a wide range of possible situations, thus more comprehensively capturing potential uncertainties in complex systems.

(2) Dynamic Feedback Mechanism

Bootstrap simulation can dynamically adjust the simulation process based on changes in the system’s state. For instance, during the simulation of mining operations, if unexpected geological events occur, the simulation process can adaptively adjust the mining plan or strategy to more accurately reflect potential real-world conditions.

(3) Propagation of Uncertainty

Bootstrap simulation tracks the pathways of uncertainty within the system, allowing for the assessment of its impact on final outcomes. For example, in stability analysis of mines, bootstrap simulation can model the progressive effects of geological model uncertainties on mining safety and quantify potential risks.

(4) Flexibility and Adaptability

Bootstrap simulation can adjust input parameters, assumptions, and model structures according to varying needs. For example, changes in geological conditions during mining operations may affect the choice of mining methods. Bootstrap simulation can accommodate these changes and optimize the mining plan accordingly.

(5) Robustness Evaluation

One advantage of bootstrap simulation is its ability to quantify the robustness of different strategies and scenarios. For instance, in mining economic optimization problems, various mining strategies can be simulated and assessed under different geological conditions and market changes to identify the most robust option.

**Fig 3:**
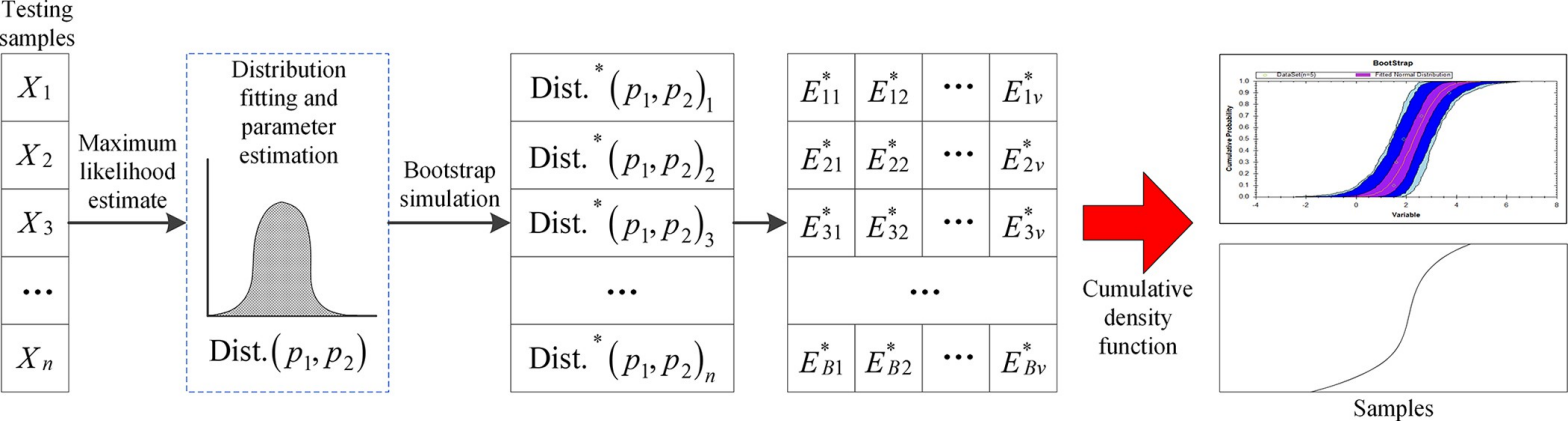
Uncertainty quantification based on bootstrap simulation.

Based on the above analysis, a quantitative uncertainty analysis using bootstrap simulation generally involves three steps, as illustrated in Fig 3 [38].

By analyzing the bootstrap samples taken by the bootstrap simulation, the uncertainty of related statistics can be described and quantified by the confidence interval or probability distribution model. Generally, a 95% confidence interval is used to describe the uncertainty range of variables. By establishing the cumulative distribution function of the relevant statistics, the values corresponding to the 2.5 and 97.5 quantiles are obtained, and the uncertainty range of the statistics (95% confidence interval) can be determined.

#### 2.4.4 Research on uncertainty transfer method

After determining the uncertainty and probability distribution functions of the input parameters, the focus shifts to studying how the uncertainty in model inputs propagates to model outputs and quantifying the uncertainty in open-pit mining revenue along with its mathematical expression. Currently, widely used methods for uncertainty propagation include the analytical method based on Taylor expansion and the Monte Carlo Method (MCM), which is based on stochastic simulation.

*2*.*4*.*4*.*1 Analytical method*. The analytical method is used to estimate the uncertainty of a model output using Taylor expansion. Assuming that *x*_1_,*x*_2_,*x*_3_,⋯ is the input parameter of the model *E* = *f*(*x*_1_,*x*_2_,*x*_3_,⋯), and the parameters are independent of each other, the output error of the model can be estimated by the next-order Taylor expansion.


$$\Delta E=|\frac{\partial f}{\partial x_{1}}\Delta x_{1}+|\frac{\partial f}{\partial x_{2}}\Delta x_{2}+|\frac{\partial f}{\partial x_{3}}\Delta x_{3}+\cdots$$ (20)

In the uncertainty analysis, the uncertainty of the input parameters and output of the model can be expressed by variance. From Eq (20), the uncertainty of the model output can be approximately divided into the sum of the parameter uncertainty contribution of each input, as shown in Eq (21):

$$Var[E]\approx {\left(\frac{\partial f}{\partial x_{1}}\right)}^{2}Var[x_{1}]+{\left(\frac{\partial f}{\partial x_{2}}\right)}^{2}Var[x_{2}]+{\left(\frac{\partial f}{\partial x_{3}}\right)}^{2}Var[x_{3}]+\cdots$$ (21)

To determine the probability distribution type and uncertainty of each input parameter, the description of uncertainty is simplified to the probability that the data fall into the confidence interval of 5%–95%, expressed by *U*, *U*∈[0,1]. According to the different combination forms of parameters in the model, the transfer of uncertainty is divided into two cases:

(1) When the output of the model is the product of multiple input parameters, the uncertainty *U*_total_ can be calculated by the sum of squares of the uncertainty *U*_*i*_ of each input parameter.


$$U_{total}=\sqrt{U_{1}^{2}+U_{2}^{2}+\cdots +U_{n}^{2}}$$ (22)

(2) When the output of the model is the sum of many kinds of input parameters, the overall uncertainty *U*_total_ can be calculated by Eq (23), where *w*_*n*_ is the influence weight of all kinds of input parameters on the output results.


$$U_{total}=\frac{\sqrt{U_{1}^{2}w_{1}^{2}+U_{2}^{2}w_{2}^{2}+\cdots +U_{n}^{2}w_{n}^{2}}}{\left|w_{1}+w_{2}+\cdots +w_{n}\right|}$$ (23)

The analytical method quickly and accurately calculates the uncertainty of the model output in some special situations. However, the analytical method is difficult to apply to the uncertainty calculation of complex models.

*2*.*4*.*4*.*2 Monte Carlo method (MCM)*. The MCM is a stochastic simulation method. Its basic principle is that the probability of events can be estimated by a large number of sampling statistics. In the general application of uncertainty analysis, the MCM randomly generates a set of independent data as model input according to the probability distribution of model input parameters and then obtains a set of model output data by corresponding numerical simulation. Finally, the output data are extracted and fitted, and the model input uncertainty is transferred and quantified.

Accordingly, because the uncertainty of the total cost is caused by the uncertainty of each sub-cost, and the uncertainty range of each sub-cost is mostly less than 60%. Therefore, the calculation result of using the analytical method to measure the overall uncertainty is more accurate. The analytical method is used to calculate the overall uncertainty in this paper.

### 2.5 Calculation of total mine revenue under uncertainty

#### 2.5.1 Quantitative study on uncertainty of coal price series

**Fig 4:**
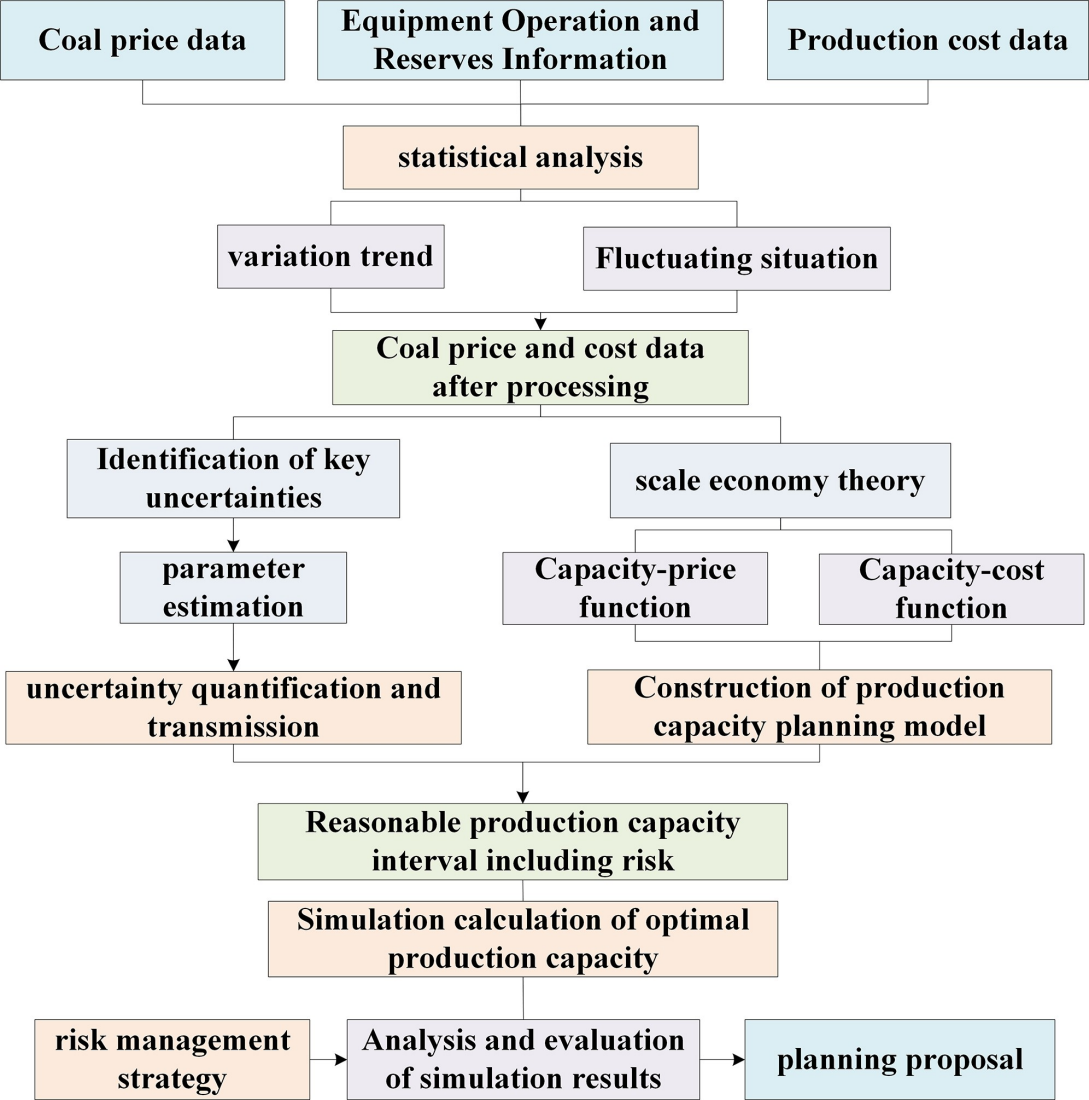
Original and predicted time series of final coal price.

To improve the calculation efficiency, this paper focuses on coal sales revenue and total production cost, ignoring other parameters that affect revenue. As a strong correlation exists between the current coal output and the previous coal price, and the coal price has obvious characteristics of changing with time, the change in coal price can be predicted by a time series analysis. The Autoregressive Integrated Moving Average Model (ARIMA) is the most used time series model in predictive analysis and is mainly controlled by three indexes: autoregressive terms *p*, moving average terms *q*, and difference times *d*. The AR(*p*) model is a *p*-order autoregressive model, and the MA(*q*) model refers to the *q*-order moving average model. When the data show evidence of nonstationarity, the ARIMA model of order *p* and *q* is applied as

$$y_{t}=c+n_{1}y_{t-1}+\cdots +n_{p}y_{t-p}+u_{t}+m_{1}u_{t-1}+\cdots +m_{q}u_{t-q}$$ (24)

where *y*_*t*_ is the ARMA series; *c* is the constant; *n*_1_,⋯,*n*_*p*_ and *m*_1_,⋯,*m*_*q*_ are parameters; *u*_*t*_ is the white noise; and *u*_*t*_,*u*_*t*−1_,⋯,*u*_*t*−*q*_ is the white noise error term. Jiang [39] used the autoregressive comprehensive moving average (ARIMA) model to estimate China’s coal prices, consumption, and investment from 2016 to 2030. The forecast result of coal price is presented in Fig 4.

With the help of polynomial fitting, the coal price series from 2000 to 2030 is fitted. The fitting equation is expressed as *y* = 5.11×10^-5^*x*^4^-0.31*x*^3^+625.27*x*^2^-421088.23*x*-835.93. Furthermore, the weight of the key factors affecting the production cost is determined by PCA, and the uncertainty range of the total cost is determined by the analytical method. Combining these with the probability distribution model of each element, we determine the mathematical expression and uncertainty range of the total income of the open-pit mine under the condition.

#### 2.5.2 Analysis of the uncertainty of total income

By combining time series prediction and uncertainty analysis, the functional relationship between coal price with time and its uncertainty range *y* = *f*(*x*)+*δ*_*p*_ are determined, where *δ*_*p*_ is the uncertainty range of coal price. To ensure the additivity of the formula, it is assumed that the uncertainty error has the same distribution characteristics as those of the coal price.

The total production cost is divided into material cost, labor cost, maintenance cost, power cost, and other itemized costs. By collecting the data of itemized costs changing with time, the optimal distribution type and uncertainty range are determined, and the mean and uncertainty range of the total cost are determined, that is, *y* = *μ*+*δ*_*c*_, where *δ*_*c*_ is the uncertainty error of the probability distribution model that accords with the model output.

To simplify the resource cost, we consider that the total income of the open-pit mine = (coal price—total production cost) × output. Using the above research results, the income curve function of an open-pit mine is determined, the marginal income of an open-pit mine is further determined, and the mathematical expression of marginal income under uncertainty conditions is obtained.

#### 2.5.3 Construction of production capacity planning model under uncertainty conditions

**Fig 5:**
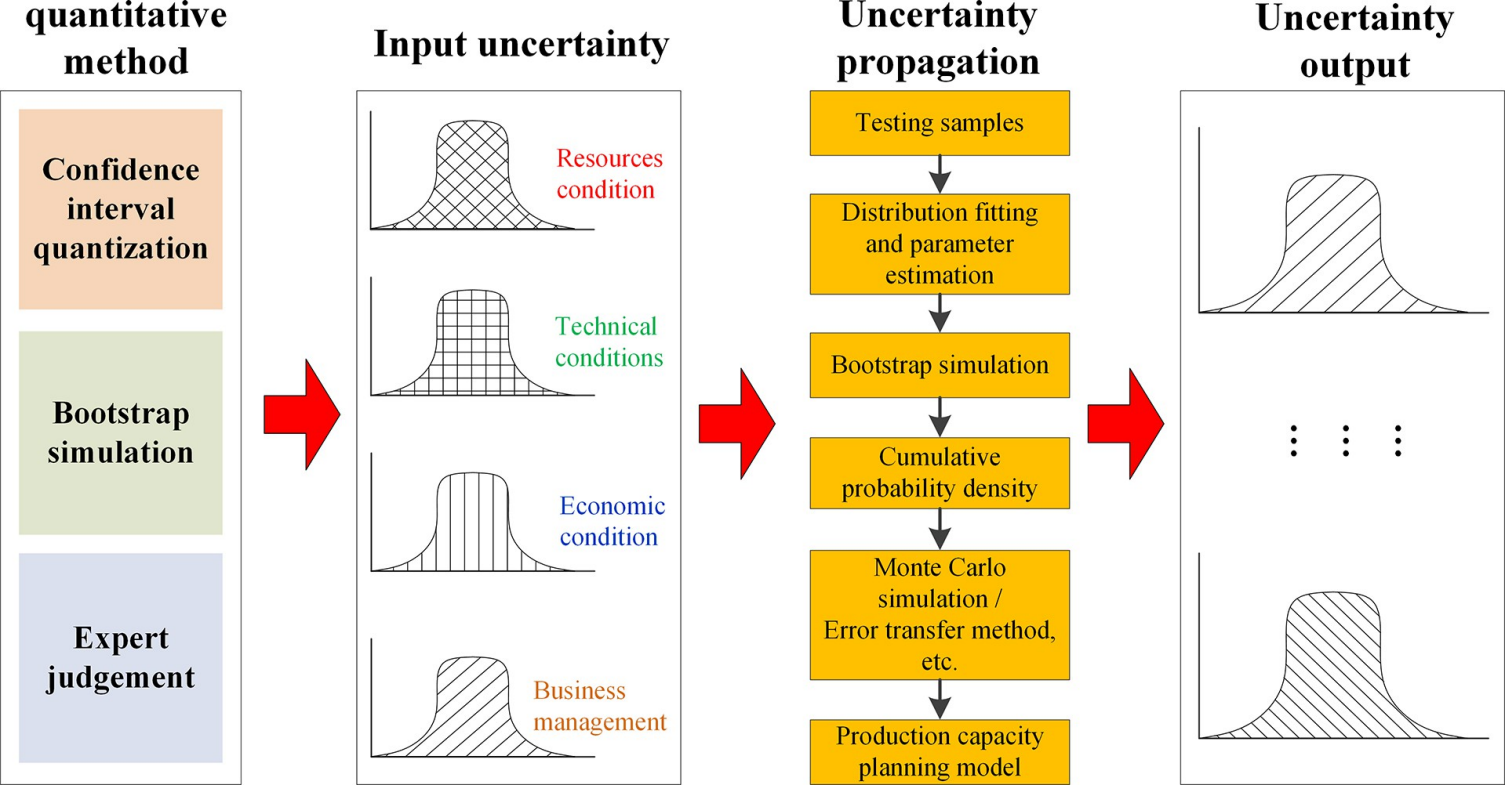
The process of determining reasonable production capacity under controlled risk.

The total revenue is consists of the determined price–output relation *P*_*t*_ = *f*(*Q*_*t*_) and the function *Q*_*t*_ = *f*(*C*_*z*_) between output and total cost. By determining the mathematical expression of income, combined with the concept of boundary income, the change trend of boundary income can be determined. Then, the reasonable productivity range of the open-pit mine can be determined using the determined maximum point. Under controllable risk conditions, the process for determining optimal production capacity is illustrated in Fig 5.

Considering that mineral price and mine cost are restricted by many factors, several kinds of uncertainty factors exist, and the superposition of many factors is further coupled into multiple uncertainties. Furthermore, the accuracy of the function fitting decreases, and the mathematical expression of the income under the condition of uncertainty is given by: $R_{Q}=P_{t}\times Q_{t}\times (1+e_{P})-C_{z}\times (1+e_{z})$. Here, *e*_*z*_ and *e*_*P*_ represent the production cost error and mineral price error caused by uncertainty, respectively. It can be seen from the expression that the uncertainty of total income is composed of two parts: coal price and cost. Both kinds of uncertainty have different effects on income. When *e*_*P*_ > 0 and *e*_Z_ < 0, the total income increases. Hence, the decision-making process disregards this part of the income as a risk. Only when the existence of uncertainty leads to a decrease in total income will the uncertainty be regarded as an economic risk to the enterprise.

**Fig 6:**
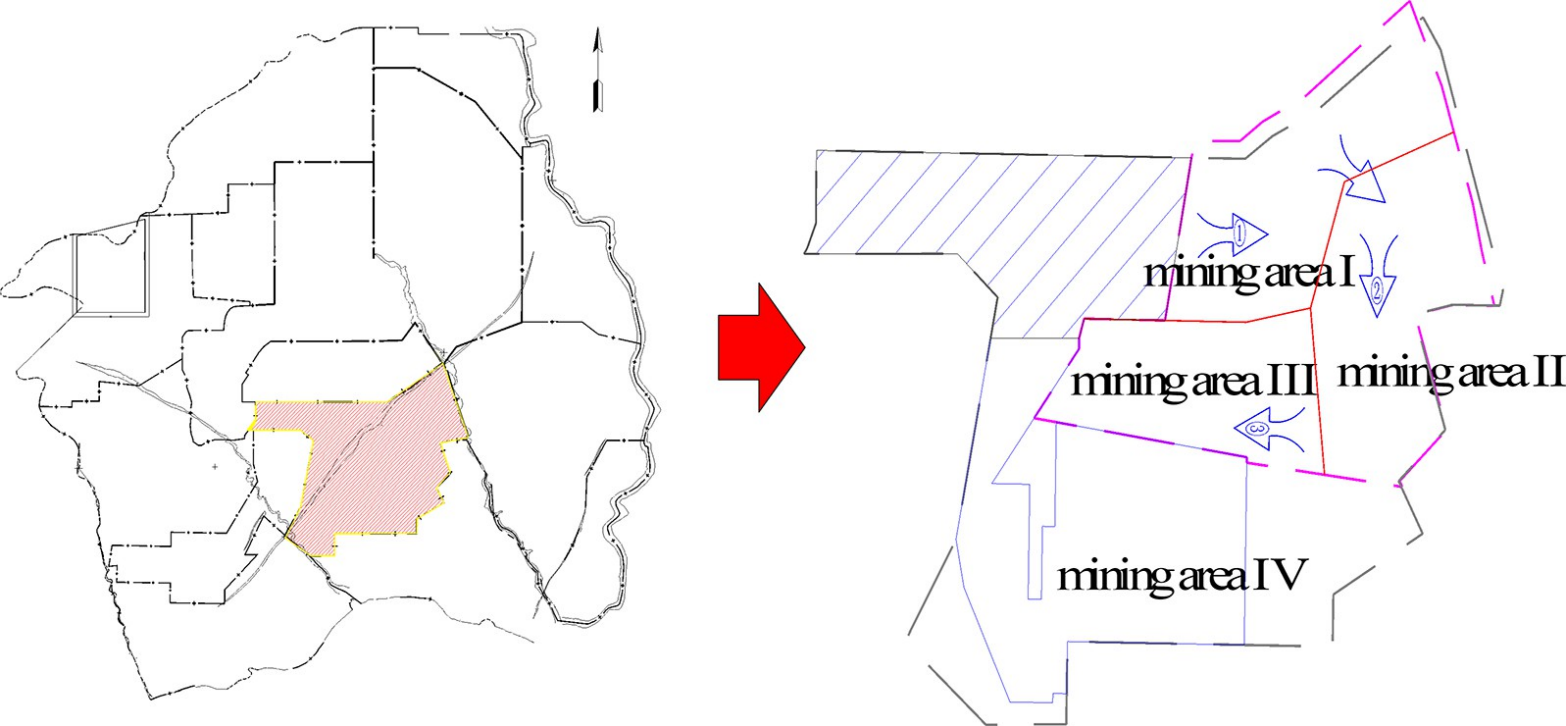
Method flow of quantitative uncertainty analysis.

To quantitatively analyze the boundary returns under uncertain conditions, it is necessary to use the observed data to describe the probability distribution characteristics of the input variables, describe the process of the transfer of the input uncertainty to the model output, and quantitatively analyze the impact of the input uncertainty on the output. The analysis process is illustrated in Fig 6.

Therefore, we perform an uncertainty analysis on the two key factors of mineral price and production cost. We also study the construction and application of an open-pit mine production capacity planning model under the condition of dual-factor coupling. The aim of this analysis is to obtain higher economic benefits under the premise of controllable enterprise risk.

### 2.6 Risk management of open-pit mine

#### 2.6.1 Risk measurement methods

For the open-pit mine, risk refers to the internal and external uncertainty risk factors existing in the entire life cycle of the project. Limited by the characteristics of risk factors, we can only make certain changes to the existing mode and conditions of risks in limited time and space. It is impossible to eliminate the risk, but the frequency of risk occurrence and the degree of risk loss can be reduced: risk can only be controlled and not eliminated.

Risk measurement is the expression of generated uncertainty by a specific numerical value. The two commonly used risk measures in the energy sector are as follows:

(1) Conditional value at risk

Given a risk level *ε* = 1 - *α* (to ensure that *ε* is meaningful, the positive part of the risk aversion coefficient is selected for analysis, that is, *α*^+^∈[0,1]; the smaller *ε* is, the lower the risk). Given a real random variable $\tilde{l}$, which represents the loss, the conditional value at risk at the *ε* level can be approximately regarded as the mean of the maximum loss in the *ε* part. The conditional value at risk CVaR*α*^*+*^ under parameter *α*^+^ is defined as follows:

$$CVaR_{\alpha }(X)=\underset{\tilde{l}\in R}{\min}\{\tilde{l}+\frac{1}{1-\alpha }E[{\left(-X-\tilde{l}\right)}^{+}]\}$$ (25)

where *X* is a random variable; (•)^+^ is a positive partial function; *α*^+^∈[0,1] is the level of risk aversion; *α*^+^ = 0 corresponds to a neutral risk (the actual value is equal to the expected value); when *α*^+^ is close to 1, the risk of the project is greater.

(2) Entropy risk measurement

If *X* is a random variable, then the entropy risk measure of *X* under the risk aversion coefficient *α*∈[−1,1] is defined as:

$$\rho_{\alpha }^{Ent}(X)=\{\begin{array}{l} \frac{1}{\alpha }logE[e^{\alpha X}]\;\alpha \neq 0,\\ E[X]\;\alpha =0. \end{array}$$ (26)

When *α*→0, $\rho_{\alpha }^{Ent}(X)=E[X]$, which indicates risk neutrality (certainty); when *α*→1, $\rho_{1}^{Ent}(X)=ess\;supX$, the decision maker tends to avoid the risk; when *α*→−1, $\rho_{-1}^{Ent}(X)=ess\;infX$, the decision maker tends to take the risk. Entropy risk is a series of convex but discontinuous risk measures, which reflect the degree of uncertainty, satisfy the additive consistency, and have good conditions for being applied to the optimization problem of an open-pit mine. The positive and negative values of *α* can be used to reflect the different risks faced by open-pit mines: when *α* < 0, decision makers tend to take risks; when *α* > 0, they tend to avoid risks.

#### 2.6.2 Determination of reasonable production capacity including risk

As previously mentioned, the economic risk caused by the change in production cost, mineral price, and other factors is the biggest risk source of open-pit mines at this stage. Usually, the economic risk of an open-pit mine includes the profit loss and cost increase that may occur in the mine, which is closely related to the fluctuation of profit indexes such as mining cost and ore selling price.

Meanwhile, mineral prices and production costs are also important factors affecting the production cost of open-pit mines, and changes in both factors will inevitably lead to risks in the process of production and operation of open-pit mines. If the risk is not managed, production according to the current production capacity may cause the enterprise to bear the risk caused by changes in economic indicators that are out of control, resulting in unbearable consequences.

The sales income and production cost of open-pit mines are two major factors that restrict the economic benefits of open-pit mines. Both factors have errors caused by uncertainty, but as they have opposite effects on income, it can be considered that the reduction in sales revenue and the increase in production costs are the main constraints leading to the income risk of open-pit mines. During the production process, the change direction of the uncertainty of sales revenue and production cost cannot be determined. Hence, it is necessary to use the uncertainty reconciliation value to determine the risk to the total income. When the sales revenue decreases, and the production cost increases, the value of the risk measurement parameter *α* is positive, and when both reach the maximum value of uncertainty, *α* is 1; otherwise, *α* is -1.

However, when *α* is close to the limit of the value range, the risk of the open-pit mine is too large to guarantee economic benefits to the enterprise according to the plan. Therefore, it is necessary to control the risk according to the actual demand of the open-pit mine. The above analysis shows that the open-pit mine income expression is *y*_*i*_ = *f*(*x*)+*δ*_*p*_−*μ*−*δ*_*c*_; when *α* is positive, the risk will occur. The income expression is divided into a time series function and an error term, and the risk is only caused by the error term. Using the above logic, we can determine the function expression of an open-pit mine income in the case of risk. Finally, the reasonable production scale under a risk controllable adjustment can be determined.

#### 2.6.3 Determination of reasonable production capacity considering redundancy

According to the definition of risk aversion coefficient *α*, its value range is [–1,1], and when *α* = 0, it is considered that there is no uncertainty. Considering the results of the uncertainty analysis, we consider that when the change in the index is within the upper and lower limits of uncertainty, the risk aversion coefficient can be taken as ±1, and the abnormal fluctuation of the data can be further considered. When the uncertainty exceeds the upper and lower limits, the limit value is taken as ±1. Meanwhile, considering the problem of redundant design, it is necessary to create room for the adjustment of production capacity, and it is considered that the capacity will be adjusted only when the change in economic indicators exceeds a certain range. For example, suppose that when the change in coal price does not exceed the range of (-5%, +5%), the production capacity requires no adjustment, that is, when the change of coal price is in the range of (-5%, +5%), the value of *α* is 0. Thus, the determination of the risk aversion level of coal price and total cost is described below.

(1) Coal price due to the uncertainty range of coal price is (-10.79%, +10.79%). To simplify the explanation process, we assume that when the change in coal price exceeds 5% of the current coal price, the production capacity will be adjusted (the determination of this range is a complex process involving historical data analysis, economic analysis, and other factors, which are ignored because they are beyond the scope of this study). In summary, the size of *α* is expressed by the following formula:

$$\alpha_{P}=\{\begin{array}{c} \frac{U_{0}-U}{U_{U}-U_{0}},U_{0}\leq U<U_{U}\\ 0,-U_{0}<U<+U_{0}\\ \frac{U-U_{L}}{U_{0}-U_{L}},U_{L}<U\leq U_{0} \end{array}$$ (27)

where *U*_0_ is custom redundancy interval; *U*_*U*_ is coal price uncertainty upper limit with a positive value, taken as +10.79% here; *U*_*L*_ is coal price uncertainty lower limit with a negative value, taken as -10.79% here. When the coal price rises, *α* is negative, and decision makers tend to take risks to obtain greater returns. When the coal price falls, α is positive, and risks tend to be avoided to reduce possible losses.

(2) Total cost

Because the uncertainty of the total cost of the mine is (-13.05%, +27.68%), it is assumed that the production capacity will not be adjusted until the change in the total cost exceeds 5% of the current cost. At this point, the formula for calculating the value of *α* is

$$\alpha_{P}=\{\begin{array}{c} \frac{U-U_{0}}{U_{U}-U_{0}},U_{0}\leq U<U_{U}\\ 0,-U_{0}<U<+U_{0}\\ \frac{U_{L}-U}{U_{0}-U_{L}},U_{L}<U\leq U_{0} \end{array}$$ (28)

where the value is positive, taken as +27.68% here; *U*_*L*_ is the coal price uncertainty lower limit with a negative value, taken as -13.05% here. When the coal price rises, *α* is negative, and decision makers tend to take risks to obtain greater returns. When the coal price falls, *α* is positive, and risks tend to be avoided to reduce possible losses. When the cost falls, the value of *α* is negative, which encourages risks to be taken to obtain greater benefits; when the cost increases, the value of *α* is positive, and risks tend to be avoided to reduce possible losses.

Knowing the risk aversion coefficient, the risk level at this time can be calculated by Eq (26). From the definition of the error, we can obtain the error $e=\frac{\rho_{\alpha }^{Ent}(X)-E[X]}{E[X]}$. Because the value range of the coal price and total cost variables is (0, +∞), the data follow the lognormal distribution. Therefore, the error also follows the lognormal distribution.

When the uncertainty error occurs, the total income of the open-pit mine with risk needs to be considered, and the marginal income of the open-pit mine under the current risk condition can be derived. Then, the changing trend of the boundary income can be determined. The maximum point is taken as the best return at this time.

#### 2.6.4 Risk management measures

Risk management refers to a scientific management method in which the units with interests related to the risk pre-estimate the potential risks that may exist in the project through risk identification, evaluation, and analysis. Reasonable and effective risk treatment measures are then proposed. Risk management is also a scientific management method for achieving the maximum project benefits at the lowest risk costs.

The size of the expected revenue from mining is directly determined by mineral prices and production costs, which in turn influence the determination of open-pit mine production capacity through economies of scale. Therefore, it is crucial to elucidate how uncertainties in these factors affect the determination of production capacity. Uncertainty in mineral prices arises from market demand, changes in the economic environment, and policy impacts, while uncertainty in production costs stems from equipment costs, labor costs, and fuel prices. Significant fluctuations in mineral prices can affect profit forecasts, thus influencing production planning. Similarly, uncertainties in production costs may lead to budget overruns and impact operational decisions. To mitigate the impact of price and cost uncertainties on production capacity determination, the following measures can be implemented:

(1) Utilize Real Options Analysis:

Adjust production strategies flexibly based on market conditions, increasing production when prices rise and reducing production when prices fall.

(2) Conduct Sensitivity Analysis of Production Costs:

Identify key factors affecting production costs and develop targeted measures in advance to address significant cost increases, thereby reducing the impact of cost changes on production capacity.

(3) Simulate Variations in Production Costs and Mineral Prices Under Different Market Conditions, this helps in formulating more robust production capacity decisions.

(4) Apply Dynamic Programming Methods:

Develop multi-stage mining production plans, adjusting production plans in phases to maximize benefits.

Adopt a strategy of risk identification, assessment, response, and monitoring for risk control. First, identify key risks using risk identification methods and assess these risks qualitatively or quantitatively using statistical methods. Next, simulate the most likely performances of various uncertainties over a future period. Finally, employ flexible production strategies to avoid, mitigate, transfer, or accept risks. Strike a balance between production capacity optimization and operational stability, incorporating dynamic adjustment mechanisms to timely adjust strategies based on actual operational conditions. Maintain optimal production amidst uncertainties to ensure long-term operational stability.

## 3. Results

### 3.1 Overview of the study area

To demonstrate the validity of the research content, this study selects a specific open-pit mine as the research subject. The open-pit mine is located in the northern part of Shanxi Province, China, with a designed production capacity of 10 Mt/a, currently expanded to 20 Mt/a. It is classified as a super-large open-pit coal mine.

Currently, the mine is in the initial extraction phase. Overburden removal is performed using a single-bucket excavator-truck intermittent process, while coal mining utilizes a semi-continuous process involving a single-bucket excavator-truck-semi-fixed crushing station-belt conveyor. Some of the stripping operations are carried out by subcontracted teams.

### 3.2 Data collection

The open-pit mine in question has been in operation for nearly 20 years, placing it in the middle to late stage of production, with coal extraction approaching its end. Due to the transition to deep mining, the mine faces challenges such as high stripping volumes, long transportation distances, high energy consumption, and poor safety conditions. To improve efficiency, reduce costs, and alleviate the direct burden on the mining company for stripping operations, the mine employs outsourcing for some of the stripping work. Given the unique and complex nature of the open-pit mining environment, outsourcing costs are influenced by factors such as workload, equipment requirements, and labor costs. Safety funds are increased due to the high-risk work environment and stringent safety regulations, while electricity costs are primarily affected by high energy consumption and power supply conditions. Consequently, these three cost components constitute a significant portion of the total production cost. Based on this, the production cost data for five months (January to May 2020) are used, as presented in Table 2.

**Table 2:** Production data sheet of an open-pit mine.

Month	January	February	March	April	May
Rock (10^4^ m^3^)	604.30	596.60	680.08	1108.45	1187.98
Raw coal (million tons)	278.69	300.23	315.09	269.04	277.29
Stripping ratio (m^3^/t)	2.22	2.41	2.31	4.23	4.37
Production cost (10^4^ yuan)					
Material costs *X*_1_	7335.03	7521.95	6782.95	7028.09	6567.49
Labor costs *X*_2_	1502.25	1105.78	944.35	1035.28	1059.05
Electricity charges *X*_3_	207.18	203.33	171.89	202.24	209.36
Water fee *X*_4_	1.27	1.49	1.36	1.39	1.60
Repair costs *X*_5_	0.00	0.00	265.73	0.00	1.04
Outsourcing stripping fee *X*_6_	910.29	711.72	1198.80	6659.56	6596.91
Equipment reverse contract fee *X*_7_	194.49	340.90	310.02	345.29	376.06
Project contract fee *X*_8_	926.15	558.72	628.46	866.88	2750.51
Other expenditures *X*_9_	171.59	162.43	222.77	93.39	132.41
Fund to maintain simple production *X*_10_	155.15	0.00	0.00	0.10	0.00
Security Fund *X*_11_	0.00	0.00	0.00	154.63	0.00

In view of the actual production of the open-pit mine, nine variable costs such as material cost and labor cost are selected. The maintenance fund and safety fund are listed as fixed costs because of their early construction time and low level of mining technology and equipment when they are put into production. Thus, the mine’s material cost, labor cost, and other expenses are high. Meanwhile, as the mine’s production is coming to an end, the maintenance fund for development is low, but the safety fund has been improved to a certain extent.

**Fig 7:**
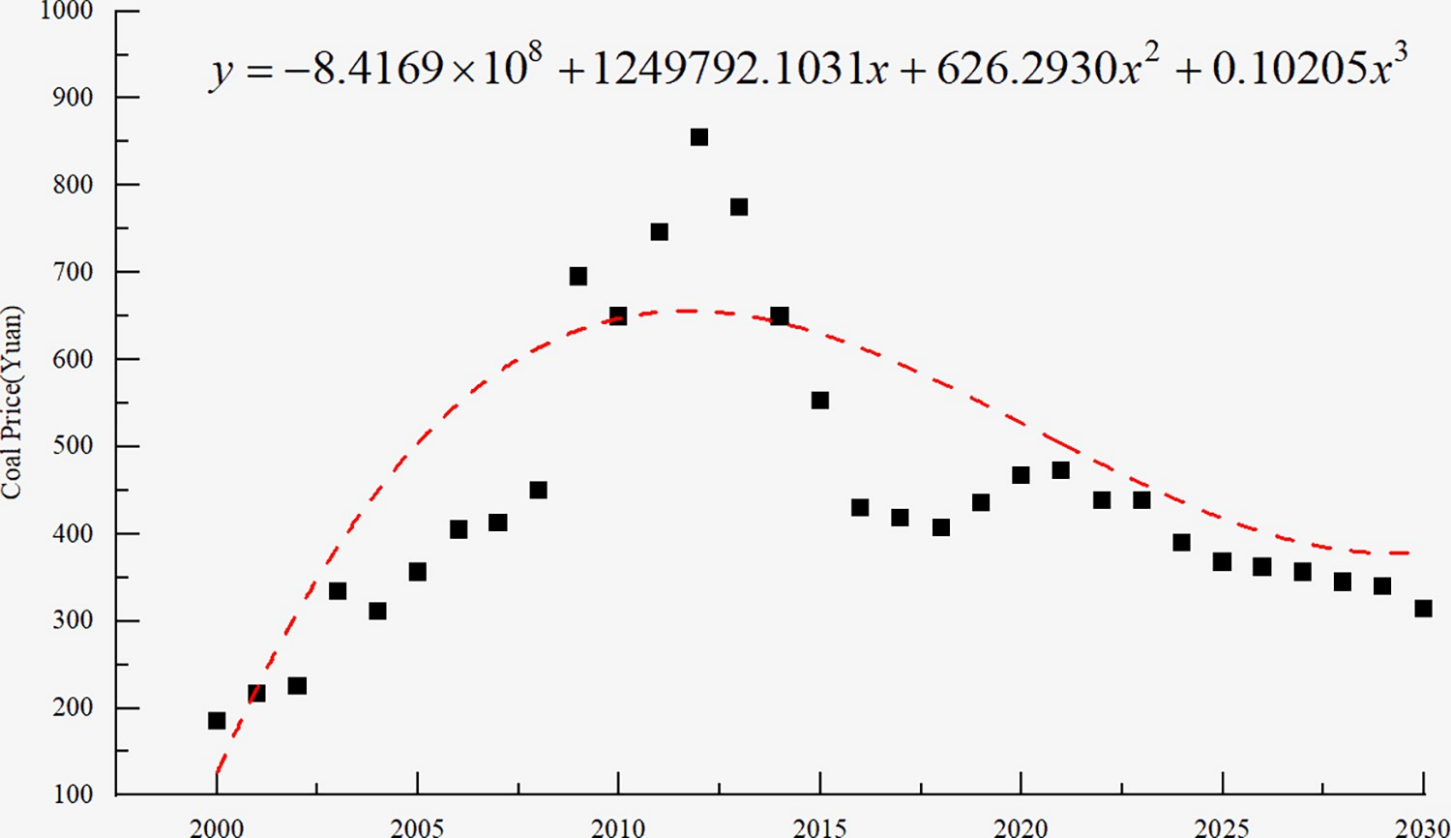
Fitting result of coal price time series.

To simplify the explanation process, we assume that the price of raw coal is consistent with the market price, that is, the coal price is only determined by market demand, and there is no direct competition between the two coal enterprises. Based on the changing trend of coal price, the curve of coal price changing with time is approximately obtained by using the polynomial fitting method, as shown in Fig 7.

### 3.3 Determination of upper and lower limits of production scale

(1) Limitation of years of service

According to the general specifications for coal mining design in China, the service life of open-pit mines is typically 35 years for super-large, 30 years for large, 20 years for medium, and over 10 years for small mines. As the open-pit mine considered in this study has been exploited for nearly 20 years, the amount of coal in the boundary should meet the mining demand for the next 15 years. As of 2020, the recoverable coal quantity within the boundary of the open-pit mine was close to 740 Mt, and when the reserve coefficient is 1.1, the upper limit of its production capacity can be calculated to be 44.8 Mt/a by Eq (3).

(2) Maximum scale that can be reached according to mining intensity

Considering the current mining situation of the open-pit mine, the length of the working line is 1700 m, the annual maximum pushing progress is 300 m to 400 m, the bulk density of raw coal is 1.4 t/m^3^, the total thickness of the minable coal seam is approximately 32 m, and the coal seam mining loss coefficient and gangue mixing coefficient are 3%. The production scale of the open-pit mine determined by the mining intensity of the mining process is 26.88 Mt/a. Meanwhile, the capacity data of each production link on the site are collected: the total capacity of the piercing equipment is approximately 110 Mm^3^, the total capacity of acquisition and installation is 91.5 Mm^3^, and the total capacity of transportation is 99.5 Mm^3^. According to the average stripping ratio of 3.11 m^3^/t, the upper production capacity is 22.26 Mt/a.

(3) Minimum scale that can guarantee benefit from the mine

According to Eq (5), combined with the data of Table 3, the minimum scale that ensures a minimum benefit from the mine should be 10281.28 t/a.

**Table 3:** Production cost correlation coefficient matrix of open-pit mine.

Cost influencing factors	*X*_1_	*X*_2_	*X*_3_	*X*_4_	*X*_5_	*X*_6_	*X*_7_	*X*_8_	*X*_9_	*X*_10_	*X*_11_
*X*_1_	1.00	0.53	0.27	-0.40	-0.38	-0.61	-0.43	-0.68	0.03	0.41	-0.02
*X*_2_	0.53	1.00	0.52	-0.51	-0.48	-0.37	-0.85	-0.07	0.02	0.96	-0.24
*X*_3_	0.27	0.52	1.00	0.32	-0.98	0.37	0.00	0.46	-0.71	0.31	0.13
*X*_4_	-0.40	-0.51	0.32	1.00	-0.27	0.50	0.85	0.69	-0.34	-0.67	-0.14
*X*_5_	-0.38	-0.48	-0.98	-0.27	1.00	-0.36	-0.02	-0.31	0.77	-0.25	-0.25
*X*_6_	-0.62	-0.37	0.37	0.50	-0.36	1.00	0.61	0.66	-0.80	-0.41	0.62
*X*_7_	-0.46	-0.85	0.00	0.85	-0.02	0.61	1.00	0.40	-0.40	-0.94	0.25
*X*_8_	-0.68	-0.07	0.46	0.69	-0.31	0.66	0.40	1.00	-0.36	-0.14	-0.17
*X*_9_	0.03	0.02	-0.71	-0.34	0.77	-0.80	-0.40	-0.36	1.00	0.18	-0.74
*X*_10_	0.41	0.96	0.31	-0.67	-0.25	-0.41	-0.94	-0.14	0.18	1.00	-0.25
*X*_11_	-0.03	-0.24	0.13	-0.14	-0.25	0.62	0.25	-0.17	-0.74	-0.25	1.00

Overall, the upper and lower limits of the annual production capacity of the open-pit mine are 0.0010–22.26 Mt/a.

### 3.4 Determination of key influencing factors of production cost

Combined with the data in Table 2, the PCA method is used to analyze the influence of material cost, labor cost, electricity fee, water fee, repair fee, outsourcing and stripping fee, equipment anti-contract fee, project contract fee, other expenditure, maintenance fund, safety fund, and other factors on production cost. The correlation matrix *R* and its eigenvalues and eigenvectors are calculated, as shown in Tables 3 and 4.

**Table 4:** Eigenvalue, eigenvector, and contribution rate of correlation coefficient matrix.

principal component	*Z*_1_	*Z*_2_	*Z*_3_	Comprehensive weight
Eigenvalue	4.7312	3.4377	1.7061	
Contribution rate	0.4301	0.3125	0.1551	
*X*_1_	-0.2822	0.2122	0.2512	0.1053
*X*_2_	-0.3089	0.3742	-0.1729	0.1169
*X*_3_	0.1034	0.5081	-0.1403	0.2033
*X*_4_	0.3761	-0.0050	-0.3185	0.1618
*X*_5_	-0.1007	-0.5094	0.0058	0.0009
*X*_6_	0.3994	0.1515	0.0915	0.2333
*X*_7_	0.4199	-0.1425	0.0419	0.1871
*X*_8_	0.2968	0.1345	-0.5106	0.1697
*X*_9_	-0.2899	-0.3706	-0.2731	0.0000
*X*_10_	-0.3499	0.2829	-0.1564	0.0884
*X*_11_	0.1796	0.1362	0.6450	0.2198

As can be seen from Table 4, the eigenvalues of the first principal component *Z*_1_, the second principal component *Z*_2_, and the third principal component *Z*_3_ are 4.7312, 3.4377, and 1.7061, respectively. Their contribution rates are 43.01%, 31.25%, and 15.51%, and the cumulative contribution rate is 89.77%.

The factors positively related to the first principal component Z_1_ are outsourcing stripping fees and equipment anti-contract fees, and the factors positively related to the second principal component Z_2_ are electricity and labor costs. The factors that are positively related to the third principal component Z_3_ and have considerable influence are safety fund fees and material fees. Then, the comprehensive weights of the influencing factors are calculated, and the results are presented in Table 4. According to the analysis, the main controlling factors that have considerable influence on the open-pit mine include outsourcing stripping fees, safety funds, and electricity fee.

To manage or minimize the uncertainties associated with the above factors, the mine can take the following measures:

(1) Enhance Stripping Cost Management and Sign long-term stripping contracts with outsourcing companies to reduce cost fluctuations caused by market volatility. Decrease the proportion of outsourced stripping work, optimize the self-operated stripping workflow, and lower unit stripping costs.

(2) Strengthen Safety Training and Preventive Measures. Implement comprehensive safety training and preventive measures to reduce the likelihood of accidents, thereby decreasing expenditures on safety funds.

(3) Improve Energy Efficiency. Sign long-term electricity agreements and explore coal-power joint ventures to reduce electricity costs.

### 3.5 Uncertainty analysis of total income

#### 3.5.1 Uncertainty analysis of coal price sequence

The coal price data follow the lognormal distribution, where the mean and standard deviation of *lnx* are 6.0374, 0.3498, and 0.6065, respectively, which meet the demand of the significant level. A bootstrap simulation is used to determine the uncertainty range to be -10.79%–10.79%.

#### 3.5.2 Analysis of income uncertainty of open-pit mine

As previously mentioned, the total income of the open-pit mine = (coal price-total production cost) × output. After calculating the uncertainty of the total cost, the uncertainty range of the total income of the open-pit mine can be determined. For open-pit coal mines, because the maintenance fund and safety fund are relatively fixed, the only question is about when to invest. Hence, it can be considered that the uncertainty of the maintenance fund and safety fund is zero. Therefore, using the data in Table 3 and the uncertainty quantitative analysis method, we analyze the uncertainty of the sub-cost; the results are presented in Table 5.

**Table 5:** Cost uncertainty analysis of an open-pit mine.

Name	Distribution type	Parameter 1	Parameter 2	Mean	Uncertainty range/%
Material costs	Uniform	6371.52	7722.68	7051.34	-4.96~5.12
Labor costs	Gamma	27.19	41.53	1131.53	-15.59~15.37
Electricity charges	Weibull	13.13	206.39	119.01	-10.40~7.69
Water fee	Gamma	125.83	0.01	1.42	-7.53~7.53
Repair costs	Uniform	-152.28	258.99	53.42	-168.36~165.06
Outsourcing stripping fee	Weibull	0.95	3521.12	3593.60	-76.24~112.96
Equipment reverse contract fee	Weibull	4.22	346.19	314.02	-23.48~23.76
Project contract fee	Lognormal	6.84	0.64	1185.73	-48.08~75.06
Other expenditures	Normal	156.52	48.01	155.88	-23.49~24.49

The commonly used probability distribution model, its corresponding probability density function, and the comprehensive weight of various factors in Table 5 are combined to obtain the overall uncertainty of the cost as (-13.05%-27.68%). Finally, the functional relationship between cost and capacity is fitted, and the approximate expression is a quadratic polynomial: *C*_*z*_ = 3.919*Q*^2^−2428*Q*+386300. Meanwhile, the relationship between coal price and open-pit production capacity is fitted, and the approximate expression is linear: $P_{t}=0.0001923{\left(Q_{t}-10\right)}^{2}-41.46\;\sin\;x+304.4$.

Then, the expression of the total income of the open-pit mine is:

$$\begin{array}{l} R_{Q}=P_{t}\times Q_{t}\times (1+e_{P})-C_{z}\times (1+e_{z})\\ =(0.0001923{\left(Q_{t}-10\right)}^{2}-41.46\sin\;x+304.4)\times (1+e_{P})-\\ \left(3.919Q_{t}^{2}-2428Q_{t}+386300)\times (1+e_{z}\right) \end{array}$$ (29)

Where *e*_*P*_∈(−0.1079,+0.1079), *e*_z_∈(−0.1305,+0.2768).

The probability distribution of the total cost is analyzed, and it is considered that the total cost conforms to the lognormal distribution. Therefore, the error is also in accordance with the lognormal distribution. Through the quantitative analysis of the total income, we can draw the following conclusions: (1) the probability distribution types of different sub-costs differ considerably; (2) the greater the output is, the greater the uncertainty of the cost is, and the uncertainty of non-proprietary costs such as repair cost and outsourcing divestiture cost is considerably higher than that of proprietary cost.

### 3.6 Determination of reasonable production capacity range to mitigate risks

Suppose that at a certain time the coal price is 300 yuan/t, the aversion coefficient *α* is 0.5, the total cost is 110 million yuan, and the aversion coefficient *α* is 0.2. Then at this time, *e*_*p*_ = 3.55% and *e*_*z*_ = -20.98%.

The expression of marginal return that includes risk is:

$$R_{Q}^{'}=-6.1932Q_{t}-42.9318\;\cos(Q_{t})-1918.6096$$

When the monthly production capacity is less than 2.22 Mt (capacity 26.68 Mt/a) and the growth rate is greater than 0, it is considered that the reasonable production scale of the open-pit mine is 26.68 Mt/a.

**Fig 8:**
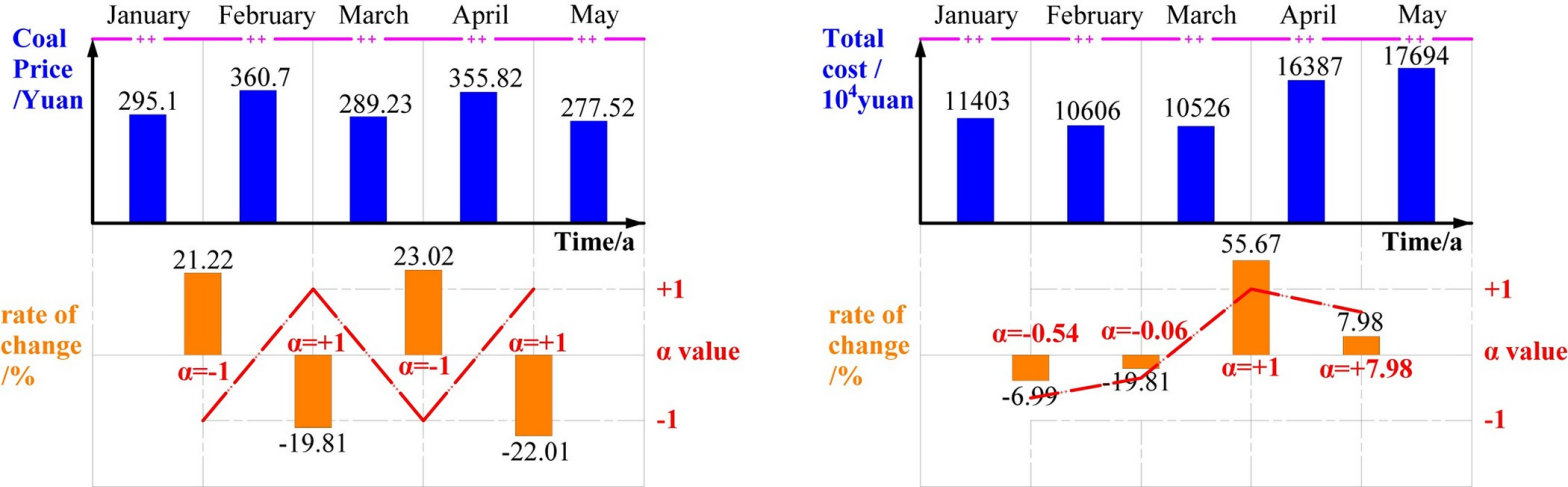
Optimal production capacity of an open-pit mine at different times.

The relationship between coal price change and cost change in the open-pit mine from January to May 2020 is determined. Taking the coal price as an example, according to the value of the coal price at two adjacent times, the change rate of the coal price is calculated, and the *α* of the position at the junction of both times is calculated by Eq (26). The entropy risk measure of the current position is also calculated, and thus the expression of the total income is obtained. Finally, the optimal production capacity at the next moment is calculated, as shown in Fig 8.

As can be seen from the left chart of Fig 8, coal price has a low uncertainty, but its variation with time is more obvious, making its *α* value constantly the extreme value. As can be seen from the right chart, except for the sharp rise in production cost from March to April, the values of *α* at other time nodes are at a more balanced level. According to the changes in *α* values at different times, the entropy risk measures of coal prices from February to May are determined to be 298.12, 356.26, 327.84 and 302.73, and the calculated errors are +0.0103, -0.0113, +0.1335 and -0.1492, respectively. Similarly, the risk measure of total cost entropy is determined to be 13975.272, 10958.27, 8391.33 and 12115.88, and the calculated errors are -0.224, -0.033, +0.2028 and +0.2582, respectively.

By substituting the above data into the marginal income expression and evaluating the expression, the monthly optimal production capacity at different times is 306.761, 306.874, 306.918 and 307.138, respectively, and the annual production capacity is 3681.132 Mt/a, 3682.488 Mt/a, 3683.016 Mt/a and 3685.656 Mt/a. The upper and lower limits of the annual production capacity of the open-pit mine are 0.0010~22.26Mt/a, indicating that the current production capacity of an open-pit mine is in a reasonable range. Moreover, greater benefits can be obtained by expanding production capacity, equipment capacity is the main factor limiting economic benefits, and a greater production capacity can be obtained by replacing equipment.

## 4. Discussion

The reasonableness of production capacity directly affects the overall economic efficiency of an open-pit coal mine. Given that production capacity planning is inevitably influenced by uncertain factors, it is crucial to simultaneously consider the practical needs for maximizing economic benefits and minimizing risks. This study is based on the theory of economies of scale and analyzes methods for determining the upper and lower limits of open-pit mine production capacity. Addressing the challenge that traditional planning methods struggle to effectively quantify uncertainties, we have constructed a production capacity planning model under uncertain conditions by analyzing the relationship between total revenue and variable factors. We argue that the presence of uncertainty leads to unavoidable risks in the capacity determination process, thus necessitating the description and quantification of uncertainties in key parameters. Furthermore, we translate the uncertainties of parameters into an expression for total revenue and use risk management techniques to determine the reasonable production capacity that incorporates risk.

Using actual production data from a specific open-pit mine, the upper and lower limits of production capacity under current mining conditions have been determined. Principal Component Analysis (PCA) identified key factors significantly affecting production costs, including outsourced stripping fees, safety funds, and electricity costs. By controlling the uncertainty of these factors, overall uncertainty can be reduced. Additionally, data on coal price fluctuations were collected, and Bootstrap simulation was used to analyze the uncertainties of coal prices and total revenue. An uncertainty propagation method was employed to determine the total revenue expression including risk, and a redundant design was considered to establish the reasonable production capacity of the open-pit mine. We verified that the current production capacity is within the reasonable range; however, due to equipment limitations, the optimal production capacity has not been achieved. This indicates that expanding production capacity remains profitable. Improving mining equipment capability could lead to higher production volumes, thereby increasing total revenue. For instance, it would allow the mine to boost production during periods of high coal demand. Furthermore, the centralization and automation levels of large equipment are generally higher, which helps improve the efficiency of individual units and reduce production costs. This approach enhances operational efficiency and overall profitability in open-pit mining by maximizing marginal benefits while minimizing production costs.

For stakeholders in the mining industry, the research findings of this study have the following practical implications:

(1) Optimizing Production Decisions: The results of this study can assist mining company managers in better understanding and addressing uncertainties related to coal prices and production costs, thereby optimizing production decisions. This helps maintain economic benefits under uncertain market conditions.

(2) Enhancing Risk Management: The research provides mining operators with powerful tools to develop effective risk management strategies. Accurate identification and assessment of these risks can inform the formulation of long-term production plans.

(3) Improving Cost Management: The methods proposed in this study help to more precisely estimate the uncertainties in production costs, thus improving cost management practices.

Compared to existing literature, the innovation of this study lies in integrating the theory of economies of scale with uncertainty analysis to construct a production capacity planning model that considers the impact of uncertain factors. This approach significantly differs from traditional open-pit mining production planning studies based on deterministic factors, as it accounts for the effects of market price fluctuations, production cost variations, and other uncertainties on production planning. While some literature has explored the impact of risk factors, there is a lack of systematic quantification and propagation of key parameter uncertainties. In contrast, this study quantifies uncertainties related to coal prices, stripping costs, and safety funds, and incorporates them into the total revenue expression, proposing a solution for optimizing production scale under uncertain conditions. By incorporating risk management into production planning, this study balances the relationship between economic benefits and risks, providing mining managers with more dynamic and precise decision support. Additionally, the research highlights that controlling key uncertainty factors can reduce overall production uncertainty, contrasting with previous studies that focused solely on addressing external market environment fluctuations. This work offers a basis for determining production capacity under uncertain conditions, provides insights and methods for analyzing cost and coal price uncertainties, and makes a valuable contribution to the determination of reasonable production capacity in open-pit mining.

Although this study provides valuable insights into production capacity planning under uncertain conditions, it is important to acknowledge its limitations:

(1) Short Time Frame and Single Mine Focus: The data used in this study spans a relatively short period and pertains to a single open-pit mine, which may not fully capture long-term trends and fluctuations, particularly in coal prices and production costs. Future research will aim to collect data over a longer time span from different regions or types of open-pit coal mines. This will help provide a more comprehensive analysis of production capacity planning performance over various time periods and enhance the generalizability of the research findings.

(2) Difficulty in Obtaining Parameter α: The acquisition of parameter α is challenging, and its importance in the model may affect the model’s usability. Future research will seek to simplify parameter settings and reduce the model’s sensitivity to parameter α. For example, exploring the use of more intuitive parameters as substitutes for α or decomposing the parameter to simplify the model structure could be potential approaches.

Overall, future research will focus on validating the proposed model under different scenarios to ensure its robustness and applicability. By developing a more generalizable model that can adapt to various uncertainties and constraints, we aim to enhance its practical value in open-pit coal mining production capacity planning.

## 5. Conclusion

Based on the theory of economies of scale, this study analyzes methods for determining the upper and lower limits of production scale in open-pit mining. It constructs a production capacity planning model for open-pit mines under uncertain conditions and translates parameter uncertainties into expressions for total revenue, using risk management to determine the risk-inclusive optimal production capacity. Analysis of actual production data from a specific open-pit mine yielded the following main findings:

(1) Method for Determining Production Scale Limits: Utilizing economies of scale theory and actual production data, a method was proposed for determining the upper and lower limits of production scale in open-pit mining. By analyzing current mining conditions, a reasonable range for production capacity was established, providing practical guidance for production planning in open-pit mining.

(2) Uncertainty Analysis of Coal Prices and Production Costs: Uncertainty factors related to coal prices and production costs were analyzed, leading to the formulation of a mathematical expression for total revenue that includes risk. The analysis of actual data reveals that uncertainties in coal prices and production costs significantly impact total revenue. This method provides a theoretical basis for production capacity planning under uncertain conditions, aiding open-pit mines in making more flexible decisions in response to market fluctuations and cost changes.

(3) Identification of Key Cost Factors: Using principal component analysis, the study identified the main factors affecting production costs in open-pit mining, including outsourced stripping costs, safety funds, and electricity costs. The research shows that controlling the uncertainties of these key factors can effectively reduce the overall uncertainty of production costs.

(4) Range of Overall Cost and Price Uncertainty: The overall uncertainty range for production costs was determined to be -13.05% to +27.68%, while the uncertainty range for coal prices was -10.79% to +10.79%. Based on this, a reasonable production capacity range was established through risk management, and it was verified that the current production capacity falls within this range. Although the current production capacity is within the reasonable range, increasing equipment capacity could still yield additional benefits from expanding production capacity.

In summary, this study presents a production capacity planning model applicable under conditions of uncertainty and verifies its practical feasibility. By controlling uncertainties in key cost factors and integrating risk management, the study provides scientific decision support for open-pit mine managers.
